# 2017 NIH-wide workshop report on “The Human Microbiome: Emerging Themes at the Horizon of the 21st Century”

**DOI:** 10.1186/s40168-019-0627-4

**Published:** 2019-02-26

**Authors:** The 2017 NIH-wide microbiome workshop writing team, Curtis Huttenhower, Gregory A. Buck, Michael P. Snyder, Owen R. White

**Affiliations:** 1 Harvard T.H. Chan School of Public Health and Broad Institute, Cambridge, MA USA; 2 Department of Microbiology and Immunology, Virginia Commonwealth University, Richmond, VA USA; 3 Department of Genetics, Stanford University, Stanford, CA USA; 4 Institute for Genome Sciences, University of Maryland School of Medicine, Baltimore, MD USA

**Keywords:** Human microbiome, HMP, NIH, National Microbiome Initiative

## Abstract

The National Institutes of Health (NIH) organized a three-day human microbiome research workshop, August 16–18, 2017, to highlight the accomplishments of the 10-year Human Microbiome Project program, the outcomes of the investments made by the 21 NIH Institutes and Centers which now fund this area, and the technical challenges and knowledge gaps which will need to be addressed in order for this field to advance over the next 10 years. This report summarizes the key points in the talks, round table discussions, and Joint Agency Panel from this workshop.

## Introduction and workshop background

In 2017, in light of the accelerating pace of human microbiome research activities and the growing support for the larger field of microbial ecology across the US federal government [1], the NIH decided to evaluate both the outcomes from the 10-year Common Fund-supported Human Microbiome Project (HMP) and, more broadly, what was needed to advance this field over the next decade. As a part of this planning, the trans-NIH Microbiome Working Group (TMWG) [2], https://commonfund.nih.gov/hmp/related_activities, which represents the 21 NIH institutes and centers that support this work through their extramural programs, recently conducted a portfolio analysis of extramural microbiome research activities over fiscal years 2012–2016 [3]. The TMWG also organized an NIH-wide workshop, “The Human Microbiome: Emerging Themes at the Horizon of the 21^st^ Century”, the subject of this report.

The workshop was organized around three themes, each spanning 1 day, with the mandate of highlighting both achievements in the field and potential next steps (Table 1). A total of ten sessions covered these themes, which comprised 43 speakers, a keynote talk for each day, and over 350 in-person attendees and 300 online attendees daily. The speakers and participants were selected from the pool of investigators in the NIH microbiome research portfolio analysis. The first day’s session opened with an overview of the HMP, including the resources it developed and its accomplishments (Mary Ellen Perry, NIH Office of the Director [2, 4, 5]), https://commonfund.nih.gov/hmp; followed by three sessions on the day’s theme “Overview and Approaches.” These sessions included talks about the state-of-the-art computational and statistical tools and animal models, as well as ethical, regulatory, and societal issues that impact this field. The second day’s theme, “Interactions of the Host-Microbiome System,” included sessions on the characterization of microbe-microbe and microbe-host interactions, the impact of diet on the microbiome and the microbiome on transforming components of the diet, and role of the microbiome in disease pathogenesis. The third day, “Microbiome Interventions for Maintaining Health and Treating Disease,” was designed to highlight the status of translational work and included two sessions that embraced a wide range of topics from the microbiome and circadian rhythm to its contributions to drug metabolism.

**Table 1:** Emerging Themes workshop themes and keynote speakers

Day	Theme	Keynote speaker	Presentation title
1	Overview and approaches	Dr. Howard Ochman	What the great ape microbiome can tell us about the human microbiome
2	Interactions of the host-microbiome system	Dr. Jeffrey Gordon	The gut microbiota and childhood undernutrition: looking at human development from a microbial perspective
3	Microbiome interventions for maintaining health and treating disease	Dr. Eric Alm	Microbiome interventions: from fecal transplants to synthetic microbial therapeutics

All speakers were asked to address one pressing technical need or knowledge gap that needs to be addressed/surmounted for the field to progress. For this report, speakers also contributed one publication related to the topic of their presentations. The workshop was structured to encourage participants to articulate and debate these gaps in knowledge and technologies through question and answer sessions and daily round table discussions. A workshop website [6], https://commonfund.nih.gov/hmp/meetings/emerging, provided a live videocast, Twitter hashtag (#ETmicrobiome), and email feedback, thus allowing receipt of questions from the online audience. The seven other federal government agencies that currently support or conduct human microbiome-related research (FDA, CDC, NIST, USDA, NSF, DOD, VA) and three NIH entities (Office of Research on Women’s Health, Office of AIDS Research, and National Institute on Minority Health and Health Disparities) participated in a Joint Agency Panel on the last day of the workshop to identify agency-specific issues as well as common themes regarding challenges to progress in this field. The talks, round table discussions, and Joint Agency Panel can be viewed online [6].

### Goals of microbiome studies

A wide range of goals for human microbiome research was highlighted during the meeting, reflecting the field’s maturation and expansion over the past decade. Translation to the clinic—discussed in detail later on—was a pervasive theme, ranging from the regulation of current practices such as fecal microbiome transplants (highlighted by panelist Paul Carlson, FDA) to combining studies of children with undernutrition with gnotobiotic mouse models to establish causal relationships between the microbiota and disease and developing new therapeutic strategies for repairing defects in postnatal microbial community development (Jeffrey Gordon, Washington Univ. in St. Louis; [7]) to early stage work to map the lung microbiome’s role in disease (Gary Huffnagle, Univ. Michigan; [5]). Perhaps the most striking aspect of the workshop’s translational emphasis was the diversity of disciplines/areas represented: precision medicine, public health policy and population health, disease prevention and early detection, establishing proof of concept, and obtaining mechanistic characterization for novel therapeutics, were all areas discussed by multiple speakers. Likewise, the challenges in advancing microbiome science (also expanded below) were diverse, from the need for deeper, more standardized population-scale studies to an understanding of what phenotypes compare well or poorly between human subjects and animal models.

Many investigators highlighted the strain-specificity of phenotypes in the microbiome: only some strains of *Eggerthella lenta* metabolize lignans (Peter Turnbaugh, UCSF; [6]), only some strains of *Ruminococcus gnavus* are present during inflammatory bowel disease (Curtis Huttenhower, Harvard; [8]), and only some strains of *Blautia producta* were sufficient to inhibit antibiotic-resistant enterococcal growth (Eric Pamer, MSKC; [9]). The field’s need for deep physiological characterization of specific isolates from relevant host phenotypes was called out throughout the workshop, as this will allow the diverse causative agents of health outcomes to be identified and studied. Indeed, several of the in vivo (e.g., zebrafish, John Rawls, Duke Univ.; [10]) and in vitro (e.g., mini-bioreactors, Rob Britton, Baylor College of Medicine; [11]) model systems discussed during the meeting were specifically intended for better characterization of microbial physiology from microbiome-derived isolate strains. It was also noted by at least one speaker that bacterial strain collections should also include isolates recovered from isolated human populations to address characterization of diversity and functions being lost with urbanization/Westernization (Maria Dominguez-Bello, Rutgers; [12]).

A topic that spanned both basic biological understanding of the microbiome and its translational applications was the investigation of microbial substrates and metabolic products, which may serve as molecular targets and biomarkers. This included identification and characterization of bioactive microbial metabolic products, as a wide range of small molecule products are now associated with specific organisms and pathways encoded by community members: examples include triolein products from propionibacteria on the skin (Pieter Dorrestein, UCSD; [13]), human milk oligosaccharides processed in the gut (Jeffrey Gordon, Washington Univ. in St. Louis; [14]), the products generated from microbial processing of dietary and host polysaccharides (Andrew Gewirtz, Georgia State Univ.; [15]), corrinoids (Michiko Taga, UC Berkeley; [16]), and many others. The workshop also touched on broader microbial interactions with dietary compounds (e.g., Gary Wu, Univ. Pennsylvania; [17] and Andrew Patterson, Pennsylvania State Univ. [18]).

Finally, several microbial mechanisms for modulating immune activity were discussed, including the role of structural variants of lipopolysaccharide lipid A during early life (Ramnik Xavier, Harvard University; [19]) and the the NOD-like receptor NLRP6 (Dana Philpott, Univ. Toronto; [20]). The discussion further underscored the importance of identifying and characterizing diverse, dynamic microbial-microbial and microbial-host interactions by integrating the technologies and concepts of different disciplines and marrying basic and translational science. As such, this field offers great opportunities for universities to develop new strategies for educating students, developing new formats for interdisciplinary centers and fostering a melding of clinical and basic sciences.

A specific illustration of the need for this cross-talk between disciplines is the need to apply quantitative modeling, engineering, and bioinformatic approaches to the microbiome. One of the greatest challenges highlighted is the microbiome’s “dark matter”—i.e., the vast numbers of genes encoding proteins of unknown function. These genes can now be readily sequenced and cataloged (Katie Pollard, Gladstone Institutes and UCSF; [21]). However, their functional contributions remain obscure and more informative methods for their annotation are needed. Another illustration relates to development of detailed metabolic models of chemical flux in microbial communities to predict the metabolic activities of communities (Elhanan Borenstein, Univ. Washington; [22]). These enzyme-/pathway-based models can, for the subset of well-annotated microbial genes/products, be linked to individual organisms and strains to identify host-specific and disease-linked differences (Curtis Huttenhower, Harvard School of Public Health; [23]). Gaps in these areas, in addition to the difficult work of expanding the base of functionally annotated microbial genes and pathways in the microbiome, include expanding the types of models capable of making molecular and phenotypic predictions and improving their robustness in the face of host-to-host and temporal variability. Synthetically modified microbes have shown therapeutic promise in laboratory settings, but developing “fool-proof” ways for regulating their activities/levels (“dose”) in vivo in different host community contexts as well as addressing governmental regulation will be formidable challenges (Timothy Lu, MIT; [24]).

### Study designs for the human microbiome and model systems

The depth and breadth of microbiome science is clearly advancing, as displayed in the presentations at this workshop. 16S rRNA gene profiling remains central to many studies, such as Howard Ochman’s (Univ. Texas, Austin) elegant use of phylogenetics, described in his keynote talk which focused on the evolution of gut microbial communities in great apes [25]. Other studies integrated a range of systems-based molecular and functional techniques, as shown by the three projects associated with the second phase of HMP, the Integrative Human Microbiome Project (iHMP), and all developed to serve as models of microbiome-associated conditions: a study to define the role of gut and nares microbiomes in development of type 2 diabetes (Michael Snyder, Stanford), an investigation of the gut microbiome in inflammatory bowel disease (Curtis Huttenhower, Harvard School of Public Health), and a study of the female reproductive tract microbiome in pregnancy and preterm birth (Gregory Buck, Virginia Commonwealth Univ.) [4]. Each of these projects integrated data from multiple technologies, including but not limited to metagenomics, metatranscriptomics, proteomics, metabolomics, immunoproteomics, and human genetic analysis. The resulting extensive datasets are available at the Data Analysis and Coordination Center at the University of Maryland (DACC) [26], https://hmpdacc.org/; preliminary analyses were described at the workshop by the projects’ representatives.

Evidence of this integrative trend continued as speakers described projects that are systems-based and multi-omic in nature. Of particular interest, shotgun metagenomic sequencing has become both affordable and analytically tractable. It has developed from a largely gene-centric exploration of microbial community diversity into a rich resource linking individual microbial strains to functional variants within a community (often including non-bacterial members). Nucleotide polymorphisms in metagenomic sequence reads, as described in several presentations, allows strain-level tracking in longitudinal samples. Eric Alm (MIT), for example, used polymorphisms to track transmission of microbial strains from FMT donors to their recipients [27]. Other speakers invoked metagenomics as a means to follow complex microbiome profiles at the subspecies level. Spatial and temporal representations of metagenomes and other ‘omics datasets were described by several investigative teams. Harris Wang (Columbia) presented a spatiotemporal metagenomics strategy to map the gut microbiome at micron scale [28]. Rob Knight and Pieter Dorrestein (UCSD) demonstrated a 3-D visualization tool to illustrate simultaneous changes in the metagenome and metabolome of the human body over time [29].

Several talks emphasized the value of having a variety of model systems to explore microbial community assembly, community member interactions, adaptive responses to perturbations, and host effects. These systems range from in vitro mini-bioreactors to phylogenetically diverse hosts (fruit flies, zebrafish, mice, pigs and non-human primates). Rob Britton (Baylor College of Medicine) has employed mini-bioreactors to culture and study complex microbial communities cost-effectively at scale [26]. Angela Douglas (Cornell) and John Rawls (Duke) pointed out the tractability of the *Drosophila* [30] and zebrafish [31] model systems, respectively, while acknowledging the challenge in transferring results that may be relevant to mammalian biology. Thaddeus Stappenbeck (Washington Univ. in St. Louis) illustrated the challenges inherent even in mouse models, which can be mitigated using carefully selected controls (e.g., littermates; [32]). Sharon Donovan (UIUC) noted the similarity of the germ-free and gnotobiotic piglet models to humans while championing their use for studying the impact of the gut microbiome on neonatal development [33].

Many, if not most, investigators have attempted to move beyond correlative associations to identify causal roles of the microbiome; they have done so by characterizing molecular mechanisms or by using a variety of perturbations, whether ecological, biochemical, environmental, host-based, or microbiological. There was a generally renewed interest in microbial culture and analysis of individual isolates and their physiology, as a way of assessing causality in a controlled environment. Andrew Goodman (Yale) established strains of bacteria with luciferase reporter genes under the control of a synthetic inducer, with the objective of manipulating specific bacteria in mice to determine the impact of perturbations on an organism in a community [34]. Many other studies were presented at the meeting; these studies included but were not limited to iHMP projects that used more than one high throughput molecular technologies to probe both host and microbe functions in attempts to characterize biochemical mechanisms that cause effects being observed.

### The diversity of microbiome data

The study designs and analyses presented at the meeting demonstrated that microbiome research is increasingly incorporating diverse culture-based, culture-independent, and molecular technologies. These approaches can capture genetic, transcriptional, translational, and metabolic features of both microbes and their hosts. It was quite evident that no single combination of technologies will apply to the diverse systems that are now of interest. Combinations of omics strategies were seen, for example, in Justin Sonnenburg’s (Stanford) integration of human dietary compounds and microbial metabolites in Western populations and in the African Hadza population [35]. This was true in many biochemically-focused sessions throughout the workshop, such as the talk by Wei Jia (Univ. Hawaii) linking liver function, bile acid production, and the neural signaling capacities of derivative compounds subsequently produced by gut microbes [36]. Several talks included multi-omic methods for longitudinal monitoring of human populations, both healthy and diseased; examples include Robert Jenq’s talk (Univ. Texas M.D. Anderson) on stem cell transplantation and graft-versus-host disease [37] as well as various NIH iHMP projects.

Conversely, it was also clear that no one assay technology, nor any particular combination, provides a “silver bullet” appropriate for tackling all study designs. Human population studies, as described above, now more typically incorporate molecular techniques that can be deployed efficiently at scale: metagenomics, metabolomics, and (decreasingly) amplicon profiling. The most detailed transcriptional, biochemical, and targeted protein quantifications were evident in model organisms and in vitro studies, albeit most often those with specific immunological targets in mind: examples include studies of antimicrobial peptides (Nita Salzman, Medical College Wisconsin; [38]) or circadian rhythms and cancer (Eugene Chang, Univ. Chicago; [39]). The welcome news is that microbiome investigators now have a broad toolbox from which to select the most appropriate combination of assays for their population, model, mechanism, and budget. The unwelcome news or reality is that technical differences can still be quite large between human microbiome protocols [40, 41] and animal models (e.g., mouse facilities; [32]).

Another emergent effect of this rich landscape of microbiome study tools is the ability to better profile non-bacterial components of the microbiome. Mahmoud Ghannoum (Case Western Reserve Univ.) discussed fungal contributions to inflammatory bowel disease [42], particularly with respect to their bacterial interactors (e.g., *E. coli* and *Serratia marcescens*). Forest Rohwer (San Diego State Univ.), conversely, called out the importance of the virome, both phage and eukaryotic viruses, and its striking diversity at the molecular (DNA/RNA, single and double stranded, enveloped, etc.) and population (inter-individual) levels. He further described the role of diverse bacteriophage as a first line of defense in the form of a bacterial-selective adaptive immune system that operates at the level of the gut mucosa [43]. In other cases, the importance of non-bacterial contributions was evident from studies of the remodeling of the gut microbiome during viral infection (Michael Snyder, Stanford). Overall, many studies have now demonstrated the utility and health relevance of pursuing each of these domain of life perspectives about the operations of microbial communities and their impact on host biology. The challenge is to apply this perspective more commonly and more systematically to the many human populations, preclinical models and/or in vitro systems which are being explored.

Likewise, surmounting the complexities encountered in integrating different data types that are incorporated into large and numerous datasets is likely to be facilitated by the changing landscape of the NIH’s management and storage of biomedical data. The NIH Big Data to Knowledge (BD2K) program was launched in 2013 to improve access to biomedical big data, and to develop and disseminate analysis methods and software [44], https://commonfund.nih.gov/bd2k. The BD2K program is now entering a second phase designed to improve cloud-based platforms where investigators can store, share, access and compute on data. The use of cloud systems for the analysis of big data is clearly required for scalability as well as to ensure access to necessary computational resources for all users. This issue was discussed in several of the workshop question/answer sessions and in the Joint Agency Panel, as well as in Owen White’s (Univ Maryland) presentation of a recent meeting designed to enable users to run microbiome analysis pipelines on cloud-based HMP data [45], http://www.igs.umaryland.edu/topics/microbiome-cloud/. This discussion demonstrated how cloud systems will allow users to analyze and share complex biomedical data, including microbiome data, with the ultimate goal of accelerating discovery and contributing to the development of microbiome-based interventions.

### Translating the microbiome

It was notable at this workshop that, even in the 10 years since the initiation of the Human Microbiome Project, great strides have been made in translating microbiome science to new therapeutics, diagnostics, and public health. The microbiome has been associated with a wide range of diseases, although what is cause and what is effect in many cases is not yet well established. The contribution of the microbiome to health, however, was evident even outside of the gut, such as in the iHMP’s project linking vaginal microbiome disruptions to complications in pregnancy and preterm birth (Gregory Buck, Virginia Commonwealth Univ.), or in the long-term consequences of mode of delivery and early life acquisition of microbial communities (Maria Gloria Dominguez-Bello, Rutgers; [46]).

Within the gut, several directions in disease investigation have now progressed far enough to link the microbiome to specific mechanisms of causation or exacerbation. The biomechanics, taxonomic diversity, and biochemistry of biofilm formation, for example, are drivers in colorectal cancer development (Cynthia Sears, Johns Hopkins Univ.; [47]). Fungal contributors are being identified (Mahmoud Ghannoum, Case Western Reserve Univ.; [48]). Also notable were several indirect routes of microbiome impact on host health, such as immunomodulation or the promulgation of antibiotic resistance or (lack of) colonization resistance (Gautam Dantas, Washington Univ. in St. Louis; [49]). Strikingly, all of these mechanistic investigations of the microbiome in disease echoed the themes of population-level studies described above: the strain-specificity of microbial phenotypes, the need to identify the right biomolecules and driving organisms, and the challenge of uncharacterized “dark matter” in different body habitat-associated communities.

The diversity of studies of the effects of diet on the gut microbiome has provided new insights about specific microbial biotransformations and how they affect nutrient harvest, immune function, as well as microbe-microbe and host signaling. For example, Nicole Koropatkin (Univ. Michigan) provided a detailed overview of the structural biology of microbial proteins encoded by polysaccharide utilization loci in Bacteroidetes; specifically how they participate in glycan sensing, acquisition and metabolism by community members and how this complex network of inter-species intra-cellular and inter-cellular transport systems is regulated with exquisite specificity to define niche, competitiveness and collaboration [44]. Polysaccharide utilization also has epigenetic consequences; Scott Bultman (Univ. North Carolina—Chapel Hill) has found that short-chain fatty acids act as histone deacetylase (HDAC) inhibitors specifically in tumor microenvironments [45]. Some study designs explicitly linked these biochemicall activities/outcomes with population-scale effects, often using meso-scale controlled feeding directly in human subjects (e.g., Johanna Lampe, Fred Hutchinson Cancer Center; [50]).

Even in this very early period of this field, the workshop highlighted the many efforts to translate the microbiome to the clinic. These efforts range from systematic analyses of donor communities for large scale fecal microbiota transplantation studies to methods for developing biotherapeutics including defined microbial consortia which are formulated in ways that reproducibly preserve their viability prior to administration. The use of host genetics as a stratification factor in population studies for individuals at risk for microbiome-associated diseases was noted numerous times. The ever expanding list of gut metabolites that may serve as diagnostic biomarkers for a range of conditions or as risk factors were also described by several speakers. These and other examples provided throughout the meeting suggested the many ways that fundamental knowledge about the microbiome will impact clinical practice in the coming years.

## Conclusions and next steps

Although the Emerging Themes workshop demonstrated that much progress has been made in human microbiome research, the field is still in its infancy with much to be learned. While the tools now clearly exist to link population-scale health phenomena to molecular mechanisms, they have been applied in an integrative fashion in only a small number of disease settings. Almost none of the conditions linked to complex dysbioses have a clear route to microbiome restoration—that is, once a perturbation is established to be causal, how can it be rationally, safely, and durably reversed or engineered to improve health? With personalized health and nutrition so much in the spotlight, it is clear that solutions will be needed to enable long-term monitoring and scientifically validated strategies for maintaining the health of our microbial communities. To further improve the benefits of personalized treatment, sex as a biological variable should be considered in all aspects of this research field (highlighted by panelist Rajeev K. Agarwal, ORWH/NIH; [51, 52]). In addition, this field, like many areas of biomedicine, is recognizing the need for broad data sharing including resources that support storage and analysis of large complex datasets, data harmonization and best practices for data management across the many and varied areas of microbiome research (highlighted in the Joint Agency Panel).

Further, despite the density and broad scope of the workshop, additional components of microbiome research and health consequences were only minimally explored. Most workshop material—and most microbiome research to date—has focused on the gut, leaving the challenges of characterizing low biomass sites and body-wide microbiome features under-emphasized. Difficult-to-study areas such as the brain-gut axis and human behavioral phenotypes also remain a challenge, as mentioned by workshop speaker Mildred Cho (Stanford) during her talk on the ethics and legal implications of microbiome research [53]. Finally, even the clearest next steps called out by the workshop—drilling down to the strain-specific molecular underpinnings of xenobiotic metabolism, immunomodulation, and dysbioses identified epidemiologically—require time, effort, and resources that have simply not yet applied in this relatively young field.

The concluding Joint Agency Panel included representatives not only from the NIH (Office of Research on Women’s Health, Office of AIDS Research, and National Institute on Minority Health and Health Disparities), but also from the NSF, CDC, USDA, NIST, FDA, VA, and DOD, all of whom called out interests in and opportunities for microbiome work at their respective agencies as well as potential collaborations between the agencies (Table 2). The panel also discussed common challenges for this field, which have been highlighted by their respective research communities and also echoed by many of the workshop speakers and participants. For example, as in the broader field of microbial ecology, the NSF and USDA noted the need for mathematical modeling and other similar approaches in microbiome research as a means of hypothesis development and testing. The CDC, DOD, and VA noted that community consensus was needed for what constituted a healthy microbiome, with the CDC proposing a Microbiome Disruption Index (MDI) as a potential metric for assessing microbiome health in future patient standard of care. NIST noted that it was still early days in this field and that common protocols for community composition analysis and interpretation were needed which would support comparative studies of the microbiome. At the other end of the research spectrum, the FDA observed there was already interest in developing microbiome-based products and devices and, as such, this agency was ready to assist this community in the regulatory process.

**Table 2:** Emerging Themes workshop Joint Agency Panel

Panelist	Agency
Dr. Rajeev K. Agarwal	Office of Research on Women’s Health, National Institutes of Health
Dr. Paul Carlson	Center for Biologics Evaluation and Research, US Food and Drug Administration
Dr. Stacy Carrington-Lawrence	Office of AIDS Research, National Institutes of Health
Dr. Linda Chrisey	Office of Naval Research/US Department of Defense
Dr. Zafar Igbal	Biomedical Laboratory Research and Development, US Department of Veterans Affairs
Dr. Scott Jackson	Biosystems and Biomaterials Division, National Insitute for Standards and Technology
Dr. Cliff McDonald	Division of Healthcare Quality Promotion, Center for Disease Control and Prevention
Dr. Jack Okamuro	Agricultural Research Service, US Department of Agriculture
Dr. James Olds	Directorate for Biological Sciences, National Science Foundation
Dr. Michael Sayre	National Institute on Minority Health and Health Disparities, National Institutes of Health
Dr. Lita M. Proctor	National Human Genome Research Institute, National Institutes of Health (moderator)

The NIH and the other federal agencies remarked that they remain committed to microbiome research in both the human and the other host habitats. The Emerging Themes workshop served to highlight the diversity of interest in this area. The NIH alone, through the TMWG, recently identified over $790M in extramural funding for the human microbiome field over FY 2012–2016 [3]. While this is enough to jumpstart remarkable infrastructure-building efforts such as the HMP, it is not enough to take full advantage of the resulting knowledge base or for the research community to fully realize the microbiome’s impact on human health. Looking ahead, federal agencies, as well as an increasing range of public-private partnerships and industry investments, will need to continue to work to fuel basic discoveries in the microbiome and, ultimately, to apply them to improve human health and disease outcomes.
